# A Case of Successful Use of the “Anchoring Technique” for Percutaneous Treatment of Left Ventricular Assist Device Graft Occlusion

**DOI:** 10.3390/ijerph19042441

**Published:** 2022-02-20

**Authors:** Rocco Edoardo Stio, Andrea Montalto, Alfredo Intorcia, Vincenzo Polizzi, Mariano Feccia, Carmine Musto, Mauro Pennacchi, Luca Paolucci, Regina Stumpo, Emilio D’Avino, Francesco De Felice, Domenico Gabrielli, Francesco Musumeci

**Affiliations:** 1 Division of Interventional Cardiology, Department of Heart and Vessels, San Camillo-Forlanini Hospital, 00152 Rome, Italy; alfredointorcia@yahoo.it (A.I.); cmusto@hotmail.it (C.M.); pennacchi.mauro@gmail.com (M.P.); l.paolucci@unicampus.it (L.P.); f.defelice1966@gmail.com (F.D.F.); dgabrielli@scamilloforlanini.rm.it (D.G.); 2 Cardiac Surgery Unit and Heart Transplantation Center, Department of Heart and Vessels, San Camillo-Forlanini Hospital, 00152 Rome, Italy; andrea.montalto@libero.it (A.M.); vpolizzi@scamilloforlanini.rm.it (V.P.); mfeccia@scamilloforlanini.rm.it (M.F.); fr.musumeci@gmail.com (F.M.); 3 Division of Vascular Surgery, Department of Heart and Vessels, San Camillo-Forlanini Hospital, 00152 Rome, Italy; regina.stumpo@gmail.com; 4 Department of Cardiovascular Anaesthesia, San Camillo-Forlanini Hospital, 00152 Rome, Italy; edavino@scamilloforlanini.rm.it

**Keywords:** left ventricular assist device, percutaneous coronary intervention, heart failure

## Abstract

Left ventricular assist device (LVAD) obstruction can be a dramatic and life-threatening complication in patients with advanced heart failure (HF). Despite surgical redo is often required in these patients, it is associated with a high risk of periprocedural negative outcome. We report the case of a 68-year-old male with a thrombotic stenosis of the LVAD proximal outflow-graft. Following Heart Team debate, a percutaneous intervention was planned during veno-arterial Extra Corporeal Membrane Oxygenation (ECMO) assistance. To achieve the needed catheter support, we used the “distal balloon anchoring technique” through the outflow-graft and managed to implant a covered stent, rapidly restoring the flow through the LVAD. The patient was discharged without further complications. Our case shows that, in selected cases, percutaneous treatment of LVAD obstructions can be feasible, especially using advanced techniques derived from the experiences in coronary interventions and under ECMO assistance. More cases and prospective studies are mostly needed to explore long-term patency of the LVADs and clinical outcomes in these high-risk patients.

## 1. Introduction

Heart failure (HF) is a common pathology, with a prevalence comprised between 1% and 2% [1]. Despite the presence of highly effective medical treatments [2], up to 5% of hospitalized patients may show advanced HF [3]. In these patients, left ventricular assist devices (LVAD) have been demonstrated to be an effective strategy and are often used as destination therapies or as bridge to heart transplantation [2]. Nevertheless, with the increasing number of patients undergoing LVAD implantation, the device-related complications are progressively increasing as well [4,5]. LVAD obstruction is a rare but potentially dramatic complication, which can be associated with high rates of mortality due to severe refractory HF. These patients are often destined to surgical redo, despite the extremely high risks related to the presence of advanced cardiac dysfunction and multiple comorbidities. In this paper, we present the case of an LVAD thrombosis causing significant outflow-graft obstruction, successfully treated with a percutaneous approach.

## 2. Case Report

**Figure 1:**
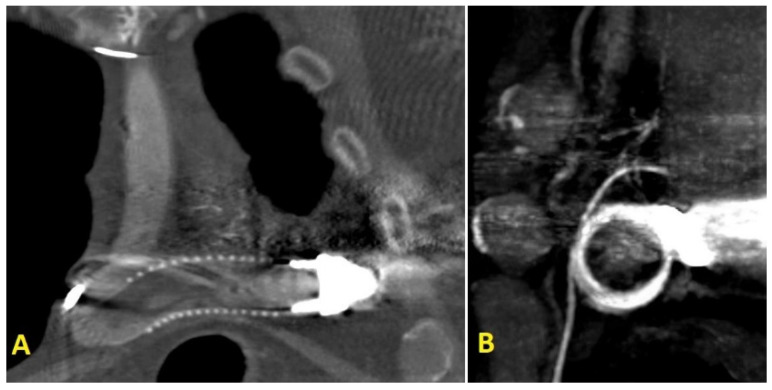
(**A**) chest CT-scan coronal-view focused on of LVAD outflow-graft; (**B**) CT-scan sagittal-view showed an image suggesting a thrombus in the proximal part of the LVAD outflow-graft.

**Figure 2:**
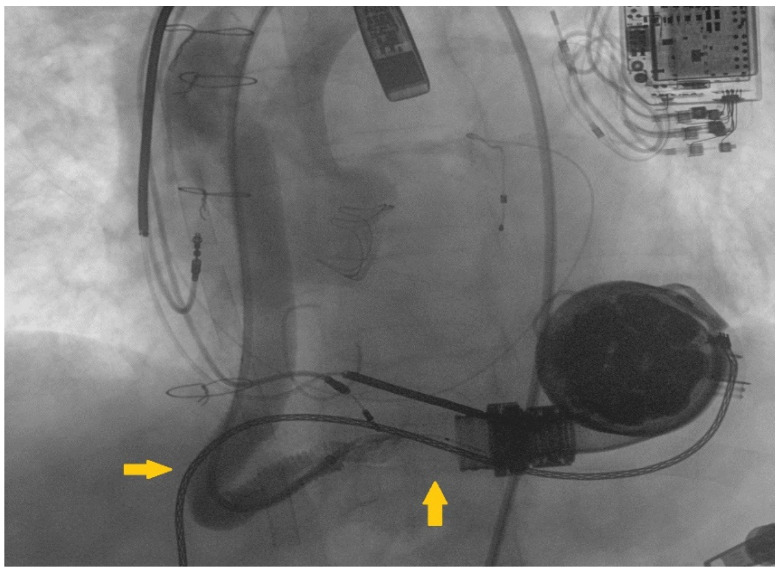
The retrograde angiography of the LVAD outflow-graft showed a kinking in the distal tract, followed by a thrombotic stenosis near the ostium of the pump (marked respectively by yellow arrows on the left and below the outflow graft).

**Figure 3:**
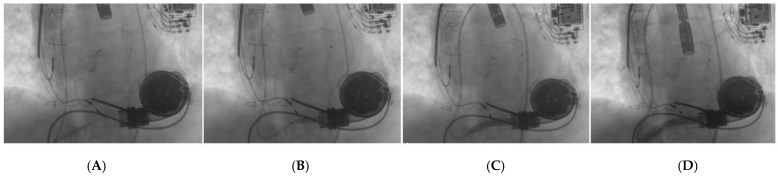
(**A**) the “distal balloon anchoring technique” was used to improve support for deliverability of balloon and stents. (**B**) balloon inflated at distal portion of the target lesion enabled us to advance the 8 Fr long sheath till the proximal part of the graft (**C**). a balloon-expandable covered-stent 10 × 59 mm was successfully implanted. (**D**) the delivery of the stent resulted in immediate evidence of blood-flow improvement through the outflow graft.

A 68-year-old male patient presented to our emergency department complaining dyspnea at rest (New York Heart Association class IV) and asthenia. Past medical history included severe aortic and tricuspid regurgitation and non-ischemic dilated cardiomyopathy associated with advanced HF. Despite being previously treated with surgical aortic valve replacement (Inspiris Resilia^TM^ 26 mm, Edwards Lifesciences LLC, Irvine, CA, USA) combined with tricuspid valve ring annuloplasty (Contour 3D^TM^, Medtronic, Minneapolis, MN, USA) no clinical benefit was obtained and the patient underwent LVAD implantation (HeartMate III^TM^, Abbott, Chicago, IL, USA) as destination therapy two years before. At clinical evaluation the patient appeared suffering and dyspnoic, with altered but relatively stable vital parameters (systemic blood pressure 100/80 mmHg, heart rate 110 beats per minute, respiratory rate 20 breaths per minute, arterial oxygen saturation 90%). A chest radiography was rapidly executed, showing severe bilateral congestion and pleural effusion. According to his past medical history, acute decompensated heart failure was suspected and an echocardiographic evaluation was requested. Transthoracic echocardiography showed a severe LV dilatation and dysfunction (ejection fraction < 25%), without significant valvular disease or pericardial effusion. Considering the lack of specific echocardiographic findings, LVAD parameters were interrogated. Despite showing a normal flow across the pump, the hemodynamic ramp test failed to reduce left ventricular end-diastolic diameter, suggesting a major LVAD malfunctioning. In order to investigate this issue further, a computed tomography (CT) was performed showing a large thrombosis sited in the proximal section of the LVAD outflow-graft (Figure 1A,B). After clinical stabilization with diuretics and inotropes, the case was object of discussion during the Heart Team session, aiming to choose the best interventional strategy for the patient. Several potential strategies were evaluated during the Heart Team meeting, including systemic thrombolysis and surgical redo. Despite thrombolysis has been proved to be effective in pump thrombosis, it can fail in treating outflow graft obstruction [6]. Following, due to the severely impaired clinical condition and the high surgical risk, percutaneous approach was considered the most appropriate first-line treatment and the patient was sent to the cath-lab. In order to guarantee hemodynamic stability during the procedure, peripheral veno-arterial Extra Corporeal Membrane Oxygenation (V-A ECMO) support was positioned advancing arterial and venous cannulas via femoral access in ascending aorta and vena cava inferior, respectively V-A ECMO flow was 5 L/min. Our first intention was to perform a retrograde angiography of the pump, aiming to evaluate the anatomi5cal setting and precise thrombosis location. Therefore, we introduced a pigtail diagnostic catheter through the right femoral artery, but we failed in reaching the proximal segments of the outflow-graft due to the presence of a severe kinking. We decided to push forward an 8Fr long sheath (90 cm Flexor^®^Ansel Guiding Sheath, Cook Medical, Bloomington, IN, USA) up to the proximal tract of the kink and performed an angiography that confirmed the presence of a severe stenosis caused by a stratified thrombosis close to the ostium of the centrifugal pump (Figure 2). In order to improve the deliverability of the balloons and stents throughout the graft and across the stenosis, we decided to use the “distal balloon anchoring technique” [7], derived from the experiences in Chronic Total Occlusion (CTO) angioplasty. Multiple inflations with peripheral-balloons of increasing diameter (from 6 × 60 mm up to10 × 50 mm MUSTANGTM 0.035”, Boston Scientific, Marlborough, MA, USA) distal to the target lesion allowed us to advance the 8 Fr long sheath close to the pump (Figure 3A,B). With the long sheath in a stable and advanced position, we managed to successfully implant a 10 × 59 mm balloon-expandable covered-stent (ADVANTA V12-8 Fr compatible covered stent, Getinge AB, Göteborg, Sweden) (Figure 3C,D), which was subsequently post-dilated. No major complications occurred during the procedure and LVAD functioning was successfully restored. Considering the stable hemodynamic profile, ECMO assistance was quickly withdrawn during the first post-procedural hours. Then, after few days spent in intensive care unit, the patient was transferred in the ordinary cardiology ward and discharged in stable conditions. Recommended therapy included a combination of vitamin K antagonist and aspirin with purpose to prevent recurrent thrombosis of both the outflow graft and the implanted stent.

## 3. Discussion

If we consider different causes of LVAD dysfunction, outflow graft obstruction appears to be a rare but potentially fatal complication, which can rapidly lead to unstable heart failure [8]. Despite major studies are critically lacking, according to data coming from the “Interagency Registry for Mechanically Assisted Circulatory Support (INTERMACS)” patients suffering LVAD pump thrombosis show significantly lower rates of survival at 1 year of follow-up [9]. Similar outcomes could be ideally expected in cases of LVAD outflow-graft thrombosis.

Even if potentially life threatening, LVAD dysfunction can be hard to identify. Indeed, considering the high prevalence of severe LV impairment in these patients, echocardiographic findings may be initially misleading. Despite being an unexplored field, 3D echocardiography could be effective in improving diagnostic accuracy in this highly complex anatomical scenario, as previously shown in other clinical settings [10]. Due to these potential delays in the diagnostic course, patients with LVAD who show acute HF of unclear origin should be referred to dedicated tertiary care, with specifically trained physicians (clinical and interventional cardiologists, cardiac surgeons, anesthetists, ect.) used to diagnose and treat advanced HF and LVAD dysfunction. Besides the diagnosis, the choice of the appropriate treatment strategy can be extremely challenging as well, and the Heart Team sessions are fundamental to evaluate each case separately. Despite surgical redo is often required in these patients, it can be associated with an unacceptably high peri-procedural risk in certain cases. In these selected cases, to reduce this risk, percutaneous approach can be chosen with the purpose to rapidly restore LVAD flow [4], especially in unstable patients.

Successful interventions in this clinical setting have been previously reported. In a recently published work, Wood et al. reviewed 26 cases of patients with evidence of outflow-graft obstruction treated with percutaneous approach [11]. In their paper, authors reported good rates of procedural success with more than 95% of patients discharged alive following the procedure. Nevertheless, despite the limited sample size of this study, mortality at 90 days seems to be critically elevated (up to 10%) [11].

Endovascular treatment of LVAD obstruction is still an unexplored field, often associated with highly challenging anatomical settings. As reported by Kalathiya and colleagues [7], severe kinking and thrombosis can often coexist across the outflow tract, limiting devices’ deliverability and causing major issues for operators who are attempting to perform a percutaneous recanalization. The use of different techniques derived from percutaneous coronary interventions can be fundamental in achieving procedural success [12], as in our case.

Patients with LVAD dysfunction are often characterized by unstable conditions, which can further hinder the achievement of procedural success. Nevertheless, it should be noted that the presence of clinical instability and the immediate need for rapid LVAD flow restoring are often considered as indications supporting percutaneous treatment instead of a surgical redo. In such cases, ECMO assistance may be crucial in order to guarantee the necessary hemodynamic stability and to avoid negative outcomes in the peri-procedural period. In our reported case, the Heart Team members considered both percutaneous treatment and ECMO assistance necessary. This should be intended as a unique and comprehensive strategy of intervention, specifically tailored on the patient’s profile.

Overall, we do think that few take-home messages should be highlighted from our specific case. First, patients with LVAD dysfunction may be critically compromised and should be rapidly referred to dedicated tertiary care centers. There are no specific clinical signs associated with this condition and patients commonly show the symptoms of acute HF. The past medical history of advanced LV dysfunction and the coexistence of many different comorbidities may be highly misleading, delaying the final diagnosis. Following, LVAD parameters interrogation and CT should be rapidly performed to confirm or exclude LVAD thrombosis in similar cases. Second, Heart Team should play a core role in selecting the best intervention strategy, once the diagnosis has been made. Each treatment (surgical or percutaneous) is ideally associated with specific benefits and risks, that must be carefully balanced. Third, percutaneous intervention, variably combined with ECMO assistance, can represent a valid strategy to avoid surgical re-intervention and to rapidly obtain LVAD flow restoring at the same time, especially in unstable patients. Interventional cardiologists who aim to perform this kind of interventions should be ready to face complex anatomical scenarios. Several technical skills derived from the experience in coronary intervention can be applied variably to this setting to achieve procedural success.

Many questions regarding this topic are obviously still open. Currently, no definitive evidence is available regarding long-term outcomes of percutaneous intervention in these patients. Even if several cases have been published during the last few years, observational studies are still critically missing. The lack of high-quality evidence is common when complex and rare clinical settings are considered. In future, major registries including data coming from several different tertiary care centers could provide new answers regarding this topic.

## 4. Conclusions

LVAD outflow graft obstruction is a rare and potentially fatal condition. Percutaneous treatment can be feasible in selected cases, especially under ECMO assistance, and could lead to rapid LVAD flow restoring. Complex anatomical scenarios are common in this setting and the use of specific techniques derived from coronary interventions (as the “distal anchoring technique”) can be useful in improving devices’ deliverability. Further studies are mostly needed to further explore clinical outcomes in these patients.

## Data Availability

Data regarding this clinical case can be provided by the authors following reasonable request.
